# Influences of Momentum Ratio on Transverse Dispersion for Intermediate-Field Mixing Downstream of Channel Confluence

**DOI:** 10.3390/ijerph20042776

**Published:** 2023-02-04

**Authors:** Jaehyun Shin, Sunmi Lee, Inhwan Park

**Affiliations:** 1 Department of Civil and Environmental Engineering, Gachon University, Seongnam-si 13120, Republic of Korea; 2 Department of Civil Engineering, Seoul National University of Science and Technology (SeoulTech), Seoul 01811, Republic of Korea

**Keywords:** channel confluence, momentum ratio, confluence angle, transverse dispersion, vertical shear in transverse velocity, helical motion

## Abstract

This study aims to analyze the influences of momentum ratio (*M*_r_) and confluence angle (*α*) on the transverse dispersion in an urban scale confluence channel from the numerical simulation results using the Environmental Fluid Dynamics Code model. By changing the momentum flux and confluence angle from the simulation results, the analysis focused on the relations between the vertical variations of transverse velocity and transverse dispersion. The high momentum tributary aligned the mixing interface toward the outer bank and created a strong helical motion, which transported the contaminated water along the channel bed and inflows into the recirculation zone. The high momentum ratio induced the large vertical shear in transverse velocity with a strong helical motion and increased the transverse dispersion. However, the helical motion persistence rapidly decreased as the flow reached downstream and led to a decrease in the transverse dispersion for the large confluence angle. Thus, the transverse dispersion coefficient increased with a high momentum ratio and low confluence angle, and the dimensionless transverse dispersion coefficient was in the range of 0.39–0.67, which is observed in meandering channels, for *M*r > 1 and *α* = 45°.

## 1. Introduction

**Figure 1:**
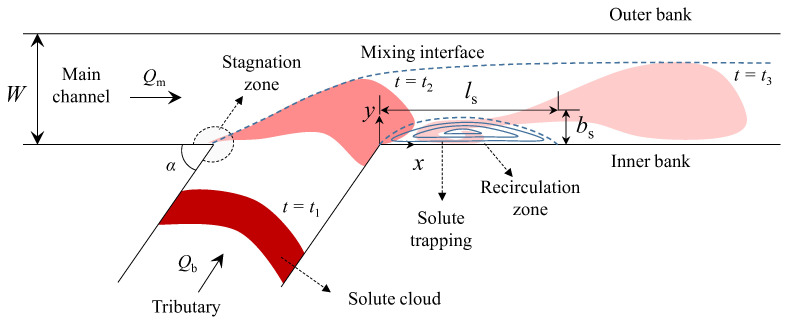
Conceptual diagrams of flow and solute mixing at channel confluence.

Tributaries crossing urban areas have the potential to be contaminated by heavy metals, road wastes, wastewater treatment plants, and other pollutant sources. The polluted tributaries are dispersed by interflowing with the mainstream channel. Therefore, hydraulic facilities such as water intake facilities and riverine parks near channel confluences are at risk from the contaminated water inflows. Thus, it is necessary to analyze the transverse mixing behavior of polluted water and its effects to control and mitigate the detrimental impacts on human health associated with these areas. However, the complex hydrodynamic properties in channel confluence cause difficulties in analyzing the polluted water behaviors. In channel confluences, the recirculation zone, mixing interface, and secondary flows, as depicted in Figure 1, appear according to discharge ratio, confluence angle, temperature differences, and channel discordance [1,2]. These flow structures, created by geometrical and hydrologic properties, influence the transverse mixing process due to the creation of the recirculation zone and secondary flows.

Previous research scrutinized the confluence hydrodynamic zone to identify the effects of tributary inflows from laboratory experiments, field observations, and numerical simulations results [1,3,4,5]. The hydraulic and geometric properties, such as momentum ratio, discharge ratio, water temperature differences, confluence angle, and bed discordance, affect the transverse velocity structures [1]. The tributary inflow forms the downwelling flow by converging with the main flow and leads to creating the mixing interface [4]. The momentum ratio plays an important role in the alignment of the mixing interface [6]. When the momentum ratio is low, the tributary momentum is too weak to penetrate the main flow, and the mixing interface is located near the inner bank. On the other hand, the strong momentum tributary inflow creates the mixing interface toward the outer bank. In the mixing interface, small eddies are developed and show coherent turbulent structures according to the momentum ratio [4]. The turbulent structures in the mixing interface influence the near-field flow and mixing processes [7]. However, to evaluate the environmental effects of reach scale water quality problems, the influences of the shear flow dispersion are more important than the turbulent diffusion.

Inward the mixing interface, the flow structures have a helical motion that promotes transverse mixing [8,9]. The mixing interface is tilted by the helical flow downstream of the confluence, and the mixing interface is realigned toward the flow with the weaker momentum flux that induces transverse mixing change [1]. The tilted mixing interface leads to producing a steep concentration gradient and promotes transverse mixing [10]. The development of a strong helical motion increases the secondary flow intensity accompanying the transverse dispersion coefficient increase [9]. The second kind of secondary flow presenting the helical motion usually appears due to the channel curvature [11,12]. The dimensionless transverse dispersion coefficient increases to the range of 0.3–0.9 in conditions of the secondary flow for a meandering channel compared to the range for a straight channel, i.e., 0.15–0.3 [13]. For considering the significance of the secondary flow, previous studies included the sinuosity or relative strength of the secondary flow to develop the empirical formulas for the transverse dispersion coefficient [14,15,16]. The helical motion induced by the tributary inflow resembles the curvature-driven secondary flows [6]. Thus, the effects of the helical motion on the transverse dispersion need to be investigated to evaluate environmental impacts by inflowing contaminated tributary. 

The purpose of this study is to evaluate the effects of momentum ratio on the transverse dispersion in downstream channel confluences. In this study, the simulation results using the Environmental Fluid Dynamics Code (EFDC) were used to evaluate the effects of the momentum ratio and confluence angle on the transverse mixing. From the simulation results in confluence channels with three confluence angles, the effects of velocity structures were discussed in the aspect of concentration-time curves formation and transverse dispersion. Furthermore, the transverse dispersion coefficients were estimated using the stream-tube moment method and the effects of the secondary flow intensity on the transverse dispersion coefficient variations were evaluated.

## 2. Theoretical Backgrounds and Methods

### 2.1. Transverse Mixing in Channel Confluences

#### 2.1.1. Mixing Properties in Channel Confluence

Mixing in confluence can be classified into slow and rapid mixing [7]. The slow mixing maintains considerable lateral concentration variations due to rapid decay in transverse flow velocity with distance downstream. Thus, the slow mixing creates a nearly vertical mixing interface, and the channel width to length for a complete transverse mixing ratio has an order of magnitude of *O*(100) [7,8]. In the case of rapid mixing, the lateral mixing completes in a relatively short distance with the persistence in vertical variations of transverse velocity and shows a tilted mixing interface. Thus, the order of magnitude in the completion of lateral mixing shows *O*(1) from past research [7,8,17].

Mixing in confluence is influenced by hydrodynamic properties (momentum ratio, discharge ratio, and density differences) of the main and tributary flows and geometrical complexities (bed elevation discordance, confluence angle, and symmetry ratio) [1]. The momentum ratio relates to the onset of the streamwise-oriented vortical cell in the mixing interface, and the high momentum ratio leads to an increase in the strength of helical motion [18,19]. The momentum ratio ($M_{r}$) is defined as follows:

(1) $$M_{r}=\frac{\rho_{b}Q_{b}U_{b}}{\rho_{m}Q_{m}U_{m}}$$

where $\rho_{b}$ is the density of tributary; $\rho_{m}$ is the density of main flow; $Q_{b}$ is the flowrate of tributary; $Q_{m}$ is the flowrate of main flow; $U_{b}$ is the cross-sectional averaged velocity magnitude of tributary; $U_{m}$ is the cross-sectional averaged velocity magnitude of the main flow. For the high momentum ratio ($M_{r}>>1$), the mixing interface is in the Kelvin–Helmholtz mode, in which the higher momentum stream aligns the mixing interface toward the outer bank and drives to grow the large-scale eddies [5]. When the momentum ratio is similar to unity, the mixing interface shows the wake mode resembling the vortex street behind an obstacle [4]. The coherent turbulent structures influence the turbulent diffusion within the mixing interface [19].

The alignment of the mixing interface influences the creation of a helical motion downstream of the confluence. The vertical profile of transverse velocity induced by the helical motion manifests to govern the transverse mixing [7,8,17,20]. The cross-sectional averaged vertical deviations of transverse velocity can be represented using the secondary flow intensity ($S_{I}$) [21], which is defined as follows:

(2) $$S_{I}=\frac{1}{m}\sum_{j=1}^{m}\frac{\sqrt{\bar{{\left({v^{\prime }}_{j}\right)}^{2}}}}{U}$$

where m is the number of measurement points along cross-section; ${v^{\prime }}_{j}$ is vertical deviations from the depth-averaged transverse velocity at the *j*th measurement point; the overbar operator, $\bar{\left(\;\;\right)}$, indicates the depth average.

#### 2.1.2. Estimation of Transverse Dispersion Coefficient

In shallow water flows, the solute mixing has been analyzed using the two-dimensional advection-dispersion equation, which can be derived from the depth average of the advection-diffusion equation:

(3) $$\begin{array}{l} \frac{\partial \left(h\bar{C}\right)}{\partial t}+\frac{\partial \left(h\bar{u}\bar{C}\right)}{\partial x}+\frac{\partial \left(h\bar{v}\bar{C}\right)}{\partial y}\\ =\frac{\partial }{\partial x}\left(h\varepsilon_{x}\frac{\partial \bar{C}}{\partial x}\right)+\frac{\partial }{\partial y}\left(h\varepsilon_{y}\frac{\partial \bar{C}}{\partial y}\right)-\frac{\partial }{\partial x}\int_{0}^{h}u^{\prime }C^{\prime }dz-\frac{\partial }{\partial y}\int_{0}^{h}v^{\prime }C^{\prime }dz \end{array}$$

where $\bar{C}=C-C^{\prime }$ is the depth-averaged concentration; C is the time-averaged concentration; $C^{\prime }$ is the vertical fluctuations of concentration; $\varepsilon_{x}$ and $\varepsilon_{y}$ are longitudinal and transverse turbulent diffusion coefficient, respectively; $\bar{u}$ and $\bar{v}$ are longitudinal and transverse depth-averaged velocity components, respectively; $u^{\prime }=u(z)-\bar{u}$ and $v^{\prime }=v(z)-\bar{v}$ are deviations of longitudinal and transverse velocity at the vertical point *z*, respectively; u(z) and v(z) are time-averaged longitudinal and transverse velocity at the vertical point *z*, respectively; h is the water depth. The last two terms on the right-hand side are the concentration flux due to the shear velocity [22]. From Taylor’s assumption, the concentration flux can be modeled using the concentration gradient and the dispersion coefficient as follows:

(4a) $$\int_{0}^{h}u^{\prime }C^{\prime }dz=-hD_{L}\frac{\partial \bar{C}}{\partial x}$$

(4b) $$\int_{0}^{h}v^{\prime }C^{\prime }dz=-hD_{T}\frac{\partial \bar{C}}{\partial y}$$

where $D_{L}$ and $D_{T}$ are longitudinal and transverse dispersion coefficients, respectively. The dispersion coefficient is regarded as the indicator to represent the dispersion by the shear flow. Thus, the dispersion coefficient influences the advection-dispersion analysis for two-dimensional solute mixing problems. For a continuous source, such as the discharge from a wastewater treatment plant and contaminated tributary inflow, $D_{T}$ plays a dominant role in the predictions of lateral mixing for a specific time and distance.

The methods for estimating $D_{T}$ are classified into the velocity-based and concentration-based methods [21]. The velocity-based method derived by Fischer et al. [22] based on the Taylor’s shear dispersion theory [23] calculates $D_{T}$ using the vertical deviations of transverse velocity as follows:

(5) $$D_{T}=-\frac{1}{h}\int_{0}^{h}v^{\prime }\int_{0}^{z}\frac{1}{\varepsilon_{z}}\int_{0}^{z}v^{\prime }\;dzdzdz$$

where $\varepsilon_{z}$ is the vertical turbulent diffusion. Equation (5) indicates that the transverse dispersion coefficient has an inverse relation with the transverse concentration gradient and a proportional relation with the concentration flux by vertical velocity deviations. The velocity-based theoretical formula can underestimate the transverse mixing compared to the concentration-based method, which includes the effects of geometrical complexities and turbulent diffusion [21]. Thus, in this study, the stream-tube moment method (STMM), which is one of the concentration-based methods, was used to estimate $D_{T}$.

The STMM was derived using the stream-tube concept under the assumption of steady-state mixing [24]. The stream-tube concept is efficient in rearranging the concentration curves for a unit discharge ($q=\int_{0}^{y}h\bar{u}dy$) passing through a cross-section. Thus, in the STMM, the concentration-time curves are treated using the dosage (θ) concept, which is defined as below:

(6) $$\theta \left(x,\;y\right)=\int_{0}^{\infty }C\left(x,\;y,\;t\right)\;dt$$

The dosage indicates the amount of concentration passing through a stream-tube. From the transverse variations of θ, $D_{T}$ is calculated using the STMM defined by:

(7) $$D_{T}=\frac{Q^{2}}{2\Psi \bar{U}H^{2}}\frac{d\sigma_{\eta }^{2}}{dx}\frac{1}{\left[1-\left(1-\eta_{0}\right)S_{1}-S_{0}\right]}$$

where Q is the flowrate; η=q/Q is the dimensionless unit discharge; $\bar{U}$ is the reach-averaged velocity; H is the reach-averaged water depth; S=θ/Θ is the dimensionless dosage; $\Theta =\int_{0}^{W}\theta \left(x,\;y\right)dy$ is the cross-sectional averaged dosage; $S_{0}$ and $S_{1}$ are the dimensionless dosage at left and right bank, respectively; $\eta_{0}$ and $\sigma_{\eta }^{2}$ are the first and the second moment of S−η curves, respectively; $\Psi =\frac{1}{Q}\int_{0}^{Q}h^{2}\bar{u}dq$ is the dimensionless shape factor. The STMM is acceptable when sufficient flow and concentration information is available [25].

### 2.2. Model Validations and Simulation Conditions

#### 2.2.1. Model Descriptions

EFDC (Environmental Fluid Dynamics Code) is the quasi-three-dimensional hydrodynamic model developed by the Virginia Institute of Technology. EFDC adopts the Reynolds averaged Navier–Stokes equation for the governing equation of flow analysis and assumes the hydrostatic condition for the momentum equation in vertical direction. The Smagorinsky model was used for the turbulence closure of the horizontal momentum equation, and the Mellor–Yamada turbulence model was employed for the vertical momentum equation. The model discretized the governing equation using the finite-difference method, and the explicit and implicit time-discretizing methods were adopted [26]. The mass transport model uses the advection-diffusion equation as a governing equation given below:

(8) $$\frac{\partial \left(hc\right)}{\partial t}+\frac{\partial \left(huc\right)}{\partial x}+\frac{\partial \left(hvc\right)}{\partial y}+\frac{\partial \left(wc\right)}{\partial z}=\frac{\partial }{\partial x}\left(h\varepsilon_{h}\frac{\partial c}{\partial x}\right)+\frac{\partial }{\partial y}\left(h\varepsilon_{h}\frac{\partial c}{\partial y}\right)+\frac{\partial }{\partial z}\left(\frac{\varepsilon_{z}}{h}\frac{\partial c}{\partial z}\right)$$

where c is the time-averaged concentration; h is water depth; u, v, and w are the time-averaged velocity components in the *x*, *y*, and *z* direction, respectively; $\varepsilon_{h}$ and $\varepsilon_{z}$ are the horizontal and vertical turbulent diffusion coefficient, respectively. The mass transport model is fully coupled with the flow analysis model. Thus, the eddy viscosity terms determined by the turbulence closure models are used as the turbulent diffusion coefficient under the Reynolds analogy assumption.

#### 2.2.2. Simulation Conditions

**Figure 2:**
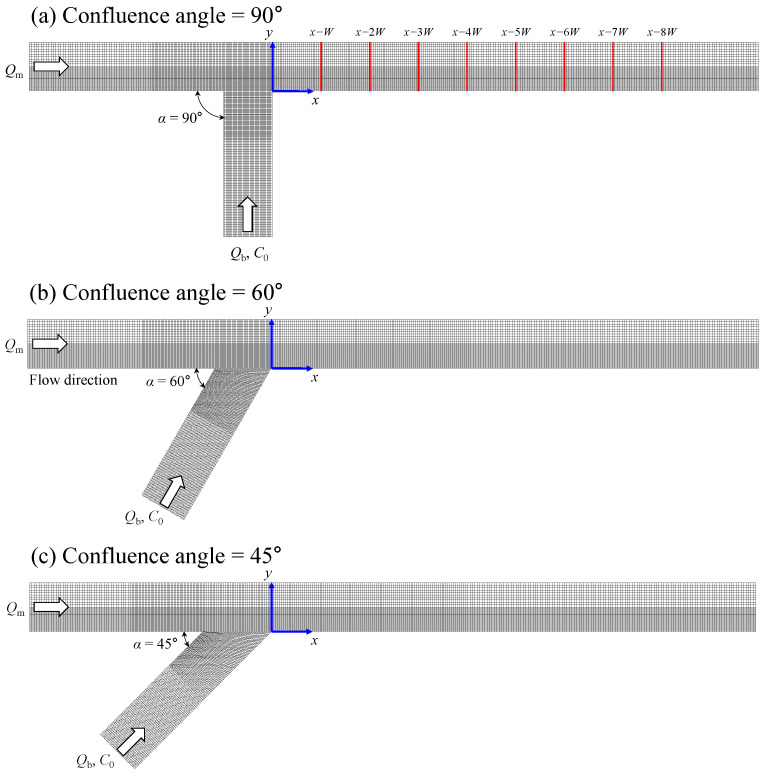
Computational mesh for simulations of solute mixing in the confluent channel.

Confluence channels with three confluence angles (*α*) = 90°, 60°, and 45° were considered for flow and solute mixing simulations. The confluence channel for α = 90° was designed based on the experimental channel by Weber et al. [27] for validation of the simulation results. For our study, the scale of the confluence channel was increased to about 10.9 times compared to the laboratory channel by Weber et al. [27], who considered small-scale urban streams paved with concrete (Manning’s *n* = 0.013) based on the Froude similarity. Thus, the channel width (*W*) is 10 m, and the width-to-depth ratio is about 3.0. The mesh size was referred to in the study by Shin et al. [9], in which the grid sensitivity was checked for the 90° confluence channel using EFDC by comparisons to the measurements by Weber et al. [27]. The study suggested the grid size as ∆*x* < *W*/20 for grid independency and computational efficiency. Figure 2 shows the computation mesh, in which the mesh size was adjusted as ∆*x* = *W*/40 − *W*/20 and ∆*y* = *W*/50 − *W*/20 for computation efficiency with acceptable computation accuracy. The number of the vertical layer was 40 (∆*z* = *h*/40) to reproduce the vertical velocity profiles near the channel confluence. For flow simulations, the upstream boundary condition was set to a constant flow rate for main (*Q*_m_) and tributary channels (*Q*_b_), and the downstream boundary condition was set with constant flow depth. The bottom and free surface boundary conditions were treated using the kinematic boundary conditions. The free-slip boundary condition was employed for the sidewall boundary condition.

Simulation conditions for this study are listed in Table 1. For neutrally buoyant water ($\rho_{m}=\rho_{b}$), the range of momentum ratio was manipulated from 0.3 to 1.8, the range of which was determined in previous research as 0.29–2.52 [1,8]. Accordingly, the discharge ratio ($Q_{r}=Q_{b}/\left(Q_{b}+Q_{m}\right)$) was 0.35–0.57. The time step was determined as 0.01 sec for satisfying the Courant–Friedrichs–Lewy (CFL) condition. The Smagorinsky coefficient (*C*_s_) for the turbulence closure of the horizontal momentum equations was calibrated as 0.2 or 0.3 from comparisons with measurements. The bottom roughness height (z_b_), which was employed to reproduce the bottom shear stress assuming a logarithmic velocity profile, was set to 1 mm for this study. The aforementioned parameters were calibrated by comparisons with the measurements by Weber et al. [27] and Yang et al. [28], which will be explained in the following section. For the solute mixing simulations, 100 ppm of solute (*C*_0_) was introduced for 5 s into the tributary channel.

#### 2.2.3. Model Validations

The flow analysis results of the EFDC program were validated by comparisons with the experimental measurements by Weber et al. [27], in which the laboratory channel has geometric similarity with the confluence channel of α = 90° shown in Figure 2a. For comparisons, the upstream boundary condition for the main and tributary channels was set as 17.03 m^3^/s and 50.29 m^3^/s, respectively, following the Froude similarity. The flow simulation results were compared with two datasets; (1) vertical velocity profiles and (2) the size of the recirculation zone.

**Figure 3:**
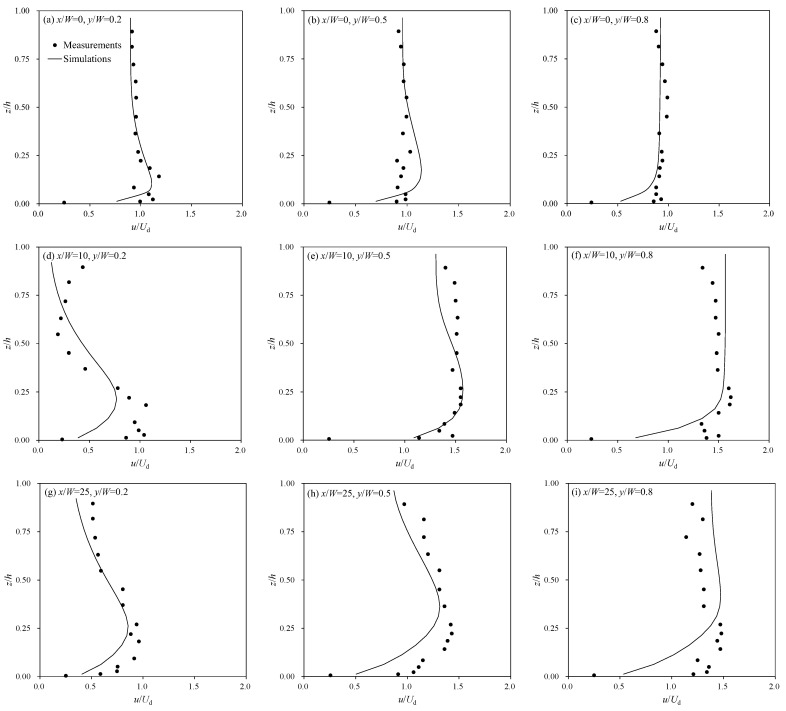
Comparisons of stream-wise vertical velocity profiles.

Figure 3 shows comparison results of the vertical profiles of the stream-wise velocity. The stream-wise velocity profiles were adequately reproduced by simulation results. However, at *x*/*W* = 1.0 and *y*/*W* = 0.2, where the recirculation zone appears, the velocity decrease near the water surface was overestimated compared to the measurements. The discrepancies occurred due to the inherent limitations of the flow analysis model, in which the vertical momentum equation was derived based on the hydrostatic condition. Thus, the simulation shows errors in the development of complex three-dimensional flow structures. Even though some differences were presented, the persistence of the velocity deficit was properly demonstrated at *x*/*W* = 2.5.

**Figure 4:**
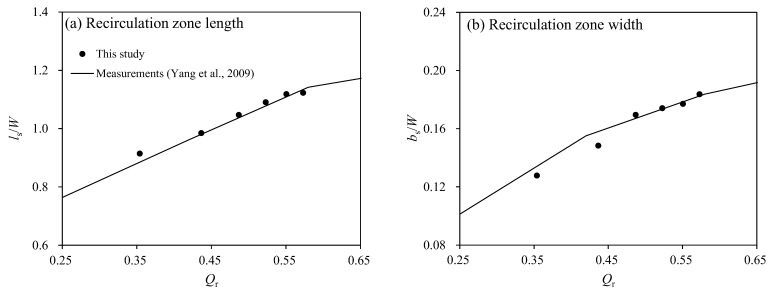
Validations of the recirculation zone size for the 90-degree confluence angle according to the discharge ratio [28].

The study by Yang et al. [28] reported the analysis results of the recirculation zone size from the experimental data of Weber et al. [27]. The study suggested a change of the recirculation zone size for the discharge ratio (*Q*_r_). Using the simulation conditions shown in Table 1, simulations were conducted and compared with the depth-averaged recirculation zone size change according to *Q*_r_ from the study by Yang et al. [28]. Figure 4 shows the comparisons between the experimental results and the simulation results. The recirculation zone’s length (*l_s_*) and width (*b_s_*) decreased when the discharge ratio decreased. The simulation results show errors of approximately 1.3% and 2.3% in the simulation results of *l_s_* and *b_s_*, respectively. Therefore, from the validation results, the model properly reproduced the flow structures in the confluence channel with α = 90°. From these results, this study assumed that the simulation results would adequately represent flow characteristics in confluence channels with α = 45° and 60°.

## 3. Results

### 3.1. Flow Analysis Results in Confluence

**Figure 5:**
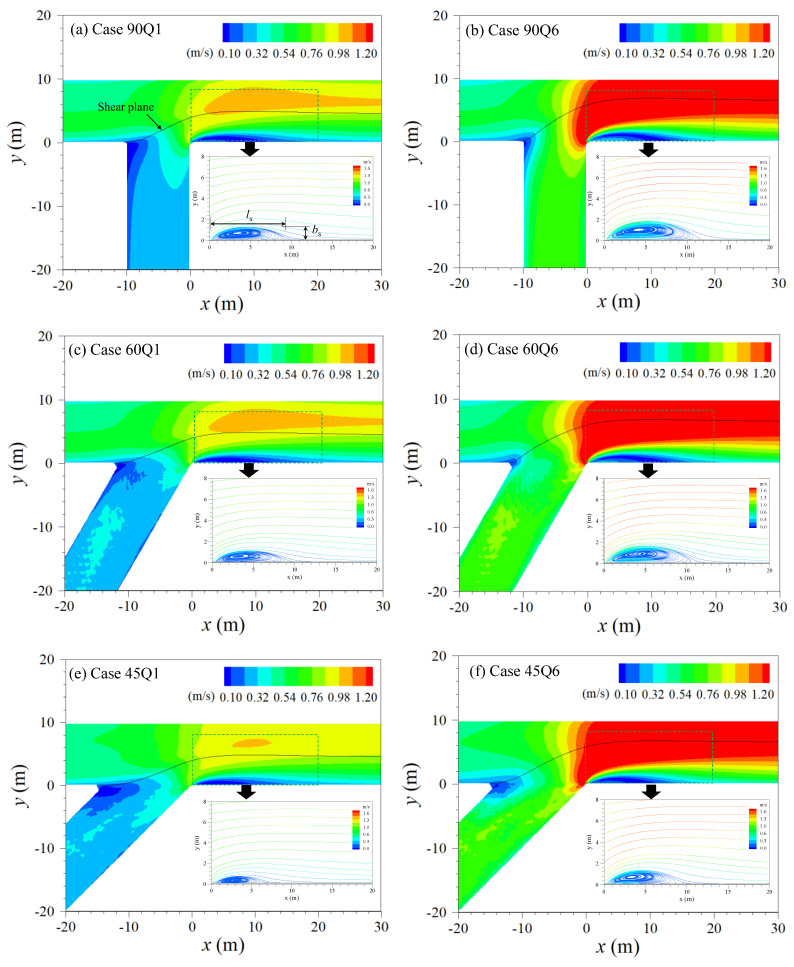
Depth-averaged velocity contours and streamlines near channel confluence.

The creation of the recirculation zone was influenced by both the discharge ratio and confluence angle, while only the discharge ratio affected the mixing interface position. Figure 5 shows the velocity magnitude and streamlines of the recirculation zone according to the change of confluence angle and momentum ratio. Inflows of tributary with high momentum (Q6) steers the mixing interface toward the outer bank (*y/W* = 1) compared to the case with low momentum (Q1), as observed in previous studies [4,6]. On the other hand, the effect of confluence angle change was insignificant to the alignment of the mixing interface, in which the mixing interface positioned at *x* = *l_s_*/2 is *y/W* = 0.68 in Case 45Q6 and *y/W* = 0.69 in Case 90Q6. These results have also been observed by Yu et al. [29], in which the mixing interface locations were similar between 30° and 90° confluence angles from the experimental studies. Related to the subject, both the length and width of the recirculation zone enlarged with the increase of discharge ratio and confluence angle, as reported in previous studies [30,31,32,33]. The enlarged recirculation zone increased the flow contraction, and the flow acceleration was more distinct when the confluence angle and momentum ratio increased.

**Figure 6:**
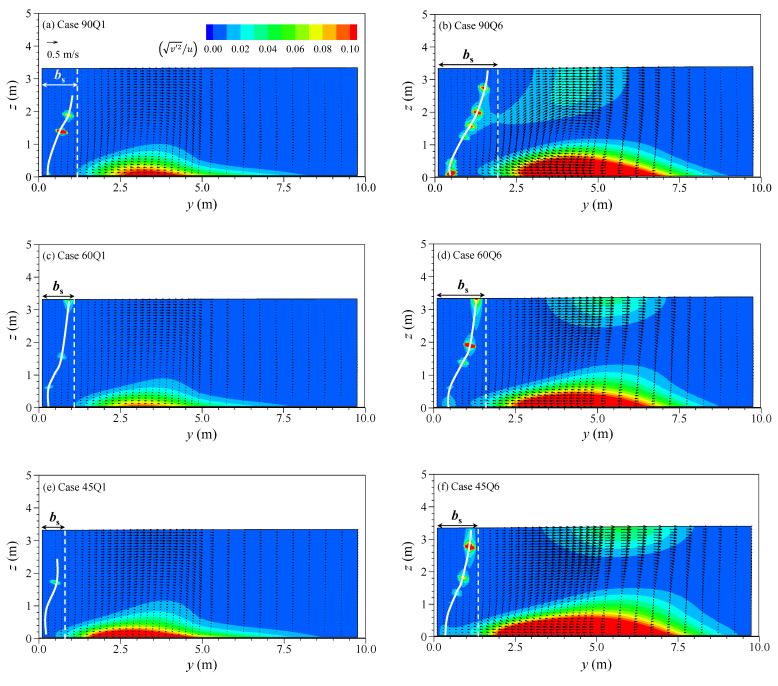
*v*–*w* vector plot with the relative strength of vertical deviations ($\sqrt{{v^{\prime }}^{2}}/u$) in transverse velocity at *x* = *l_s_*/2.

The momentum ratio mainly influences the transverse velocity structures in the center of the recirculation zone. Figure 6 shows the intensity of vertical deviations in the transverse velocity profile ($\sqrt{{v^{\prime }}^{2}}$) relatively compared to the longitudinal velocity magnitude (*u*) in the form of $\sqrt{{v^{\prime }}^{2}}/u$ and *v-w* velocity vectors at the center of the recirculation zone (*x* = *l_s_*/2). The $\sqrt{{v^{\prime }}^{2}}/u$ term can be used to quantify the size of the characteristic of the secondary current at each vertical point, which has similar characteristics as the secondary flow intensity, except it uses vertical point values instead of using cross-sectional averaged values. In the cross-section at x = ls/2, downward flow by converging two streams appears at the mixing interface, and the immersed fluid moves to the inner bank along the channel bottom, which creates one clockwise rotating circular cell. The clockwise rotating circular cell causes the fluid to flow from near the channel bed toward the recirculation zone (*y* < *b_s_*). The onset of the circular cell affects the relative vertical deviations of transverse velocity ($\sqrt{{v^{\prime }}^{2}}/u$), which increased near the channel bed and water surface. The increase in $\sqrt{{v^{\prime }}^{2}}/u$ near the channel bed is caused by the decrease in *u*, while the increase near the water surface is due to the increased transverse velocity by the tributary inflow with significant transverse momentum. In the recirculation zone (*y* < *b_s_*), a peak value of $\sqrt{{v^{\prime }}^{2}}/u$ (white line in Figure 6) appeared near the recirculation zone boundary at the water surface and the inner wall (*y* = 0 m) at the channel bottom. These patterns were consistent in every simulation case. The increase of $\sqrt{{v^{\prime }}^{2}}/u$ can be observed more clearly in the case with high momentum ratio. However, the confluence angle change rarely contributes to the increase. These results indicate that the high momentum tributary inflow dominantly drives the formation of the circular cell and the increase of transverse velocity shear downstream of the confluence.

**Figure 7:**
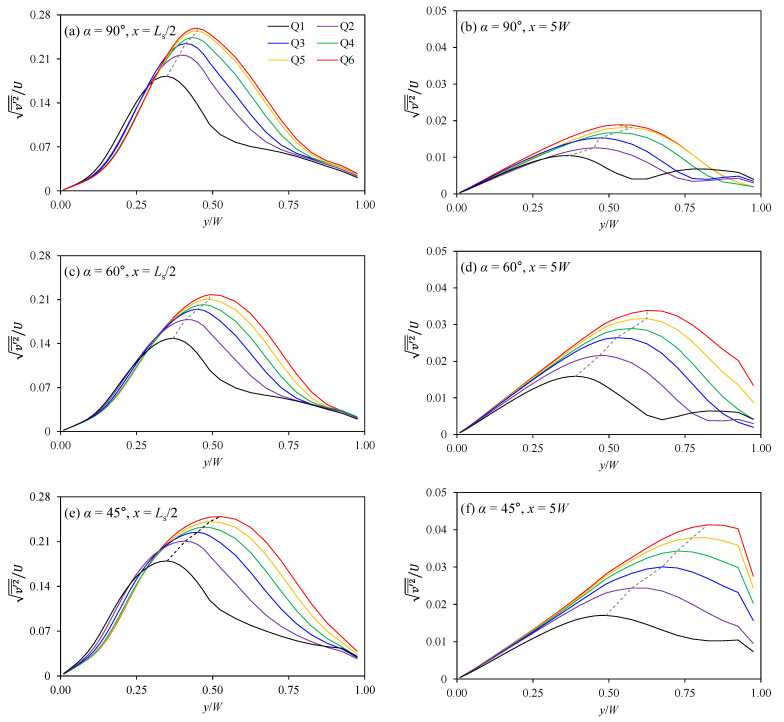
The relative intensity of transverse velocity shear against the primary velocity at *x* = *L_s_*/2 and *x* = 5*W* (dashed line: the trajectory of the maximum transverse velocity shear).

The relative intensity of depth-averaged vertical deviations in the transverse velocity ($\sqrt{\bar{{v^{\prime }}^{2}}}/U=\sqrt{\frac{1}{h}\int_{0}^{h}{\left(v\left(z\right)-\bar{v}\right)}^{2}dz}/U$) shows distinct features where the relative intensity is weakened as taken apart from the confluence and strengthened with the momentum ratio increase. The term is similar to the secondary flow intensity, but uses depth-averaged values instead of cross-sectional averaged values. Figure 7 shows the transverse distributions of $\sqrt{\bar{{v^{\prime }}^{2}}}/U$ at *x* = *l_s_*/2 and *x* = 5*W*. The values of $\sqrt{\bar{{v^{\prime }}^{2}}}/U$ decrease as the momentum ratio decrease and maintain the descent downstream of the confluence due to the gradually disappearing circular cell. The peak values of $\sqrt{\bar{{v^{\prime }}^{2}}}/U$ at *x* = 5*W* decrease to 5.7–22.6% of those at *x* = *l_s_*/2 according to the momentum ratio change. Meanwhile, as shown from the dashed line in Figure 7, the peak value is aligned toward the outer bank at *x* = 5*W* compared to that at *x* = *l_s_*/2. The decrease of $\sqrt{\bar{{v^{\prime }}^{2}}}/U$ in the downstream area is relevant as the increase in the confluence angle. For example, compared to *x* = *l_s_*/2, the peak value of $\sqrt{\bar{{v^{\prime }}^{2}}}/U$ for Case Q6 decreased to 10.3% at *x* = 5*W* in α = 45° and 5.7% in α = 90°. With a small confluence angle, the transverse momentum decrease by the merging of the main and tributary flows is being retarded, and the circular cell maintains even in farther downstream. On the other hand, the increase in the momentum ratio makes the peak value of $\sqrt{\bar{{v^{\prime }}^{2}}}/U$ increase. The inflow of tributary with high momentum creates the mixing interface accompanying a strong circular cell and increases the vertical deviations of the transverse velocity. These results indicate that both the discharge ratio and the confluence angle influence the transverse shear dispersion, which depends on the vertical velocity structures.

### 3.2. Solute Mixing in Confluence

**Figure 8:**
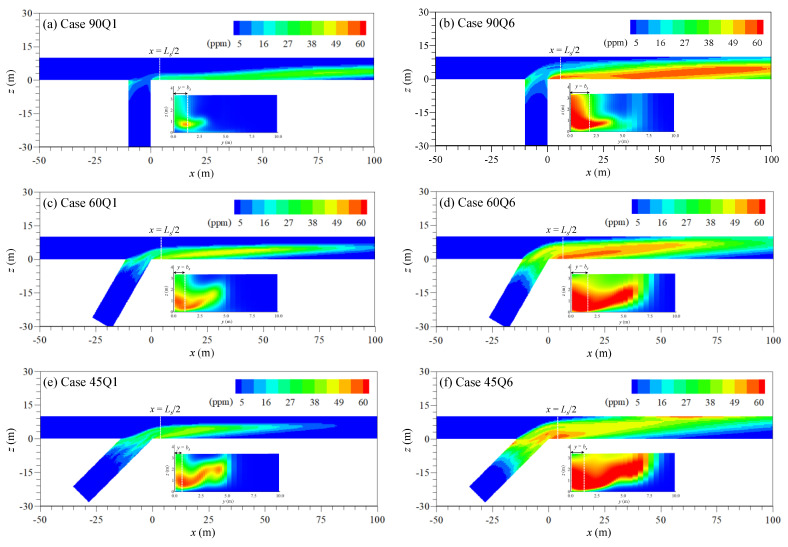
Concentration distributions when the peak concentration occurs in the recirculation zone.

The solute mixing simulation results in the confluence indicate that the transverse dispersion is promoted with the momentum ratio increase. Figure 8 shows the concentration contours from the solute mixing simulation results when the peak concentration occurs in the recirculation zone. The subplot in the figure is the cross-sectional concentration distribution at *x* = *l_s_*/2. The contaminated water from the tributary inflow is mainly transported to the downstream area along the inner bank due to the onset of the circular cell inside the mixing interface. The clockwise circular cell causes the contaminated water to enter the recirculation zone along the channel bed (see Figure 8), and the high concentration appears near the channel bottom. The upwelling flow at the recirculation zone boundary introduces the solute into the recirculation zone, and the weak transverse vertical velocity shear stagnates the solute in the recirculation zone. According to Equation (5), the transverse dispersion increases with the increase of vertical deviations of the transverse velocity, and the peak location of $\sqrt{\bar{{v^{\prime }}^{2}}}/U$ is positioned toward the outer bank as the increase of the momentum ratio as shown in Figure 7. Thus, when comparing Case Q1 and Q6, the pollutant cloud further dispersed to the outer bank in Case Q6.

**Figure 9:**
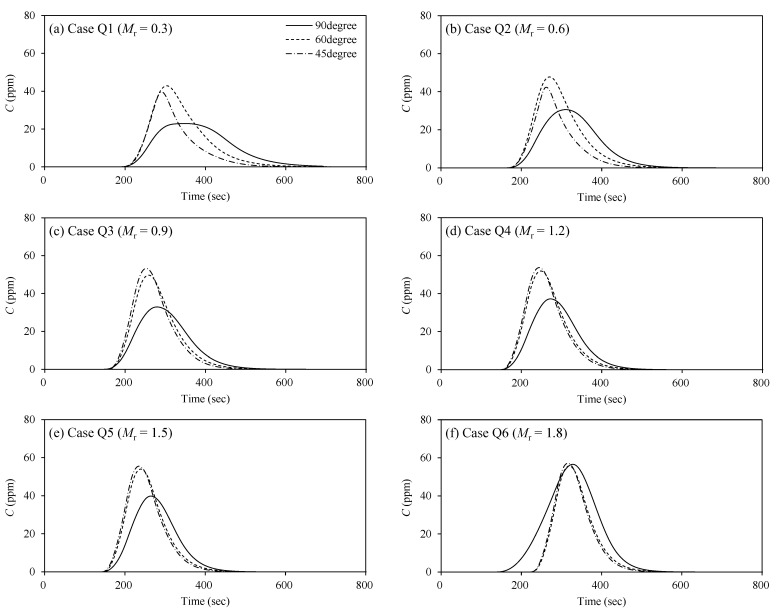
Depth-averaged concentration-time curves at the center of recirculation zone (*x* = *l_s_*/2, *y* = *b_s_*/2).

The contaminated water trapped in the recirculation zone produces the anomalous mixing that creates the non-Gaussian concentration-time curves. Figure 9 shows the depth-averaged concentration-time (*C-t*) curves at the center of the recirculation zone (*x* = *l_s_*/2, *y* = *b_s_*/2). Table 2 shows the statistical properties of *C-t* curves shown in Figure 9. In Table 2, the retention time (*t_d_*) of the contaminated water in the recirculation zone was calculated from the difference between the inflow time and outflow time of the concentration corresponding to 1% of the peak concentration (*C_p_*). The main feature of *C-t* curves in the recirculation zone is that the curves have a long tail (*ξ_t_* > 0), and the skewed shape intensifies with the decrease of the momentum ratio. The skewed concentration curves are the general feature observed in the storage zone effect [6]. At the same time, the retention time (*t_d_*) of contaminated water also increased with the momentum ratio decrease. The results indicate that the contaminated water stayed longer in the recirculation zone as the momentum ratio decreased. The clockwise circular cell transports the solute from the tributary to the recirculation zone (see Figure 6). The trapped solute is then discharged outside of the storage by the high value of $\sqrt{{v^{\prime }}^{2}}$ occurring near the recirculation zone boundary on the water surface. Thus, the tail of *C-t* curves was exceptionally reduced in Case Q6 for α = 90°, in which $\sqrt{{v^{\prime }}^{2}}/u$ increased compared to other cases.

### 3.3. Estimation of Transverse Dispersion Coefficient

**Figure 10:**
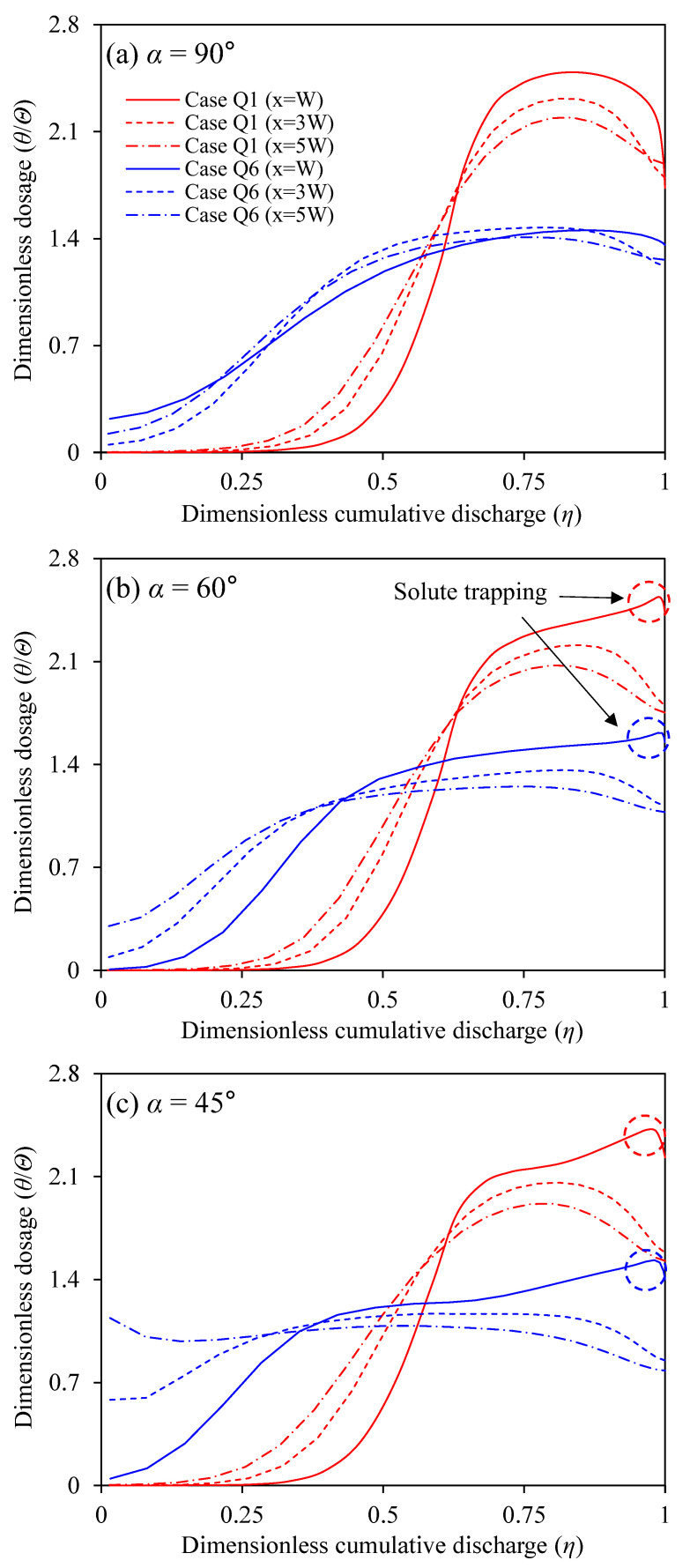
Dimensionless dosage curve variations with the discharge ratio change (*η* was calculated from the left bank (*y*/*W* = 0) to the right bank (*y*/*W* = 1), which means *η* = 0 at *y/W* = 1 and *η* = 1 at *y/W* = 0).

As the flow reaches downstream from the confluence, the dosage curves are flattened by the proceeding transverse mixing. The dimensionless dosage curves (θ/Θ) indicate the amount of contaminated water flowing through a cross-section. θ/Θ is compared in Figure 10 with the change of the discharge ratio and confluence angle at *x* = *W*, 3*W*, and 5*W*. For Case Q1, most of the solute passes through the inside of the mixing interface (*η* > 0.5 and *y/W* < 0.7). The results indicate that the primary flow accompanying the circular cell plays a dominant role in the advection of the contaminated water from the tributary. When the discharge in the tributary increases to Q6, θ/Θ curves show a flattened shape compared to those in Case Q1 due to the strengthened transverse dispersion by the inflow of tributary with high momentum. The decrease in the confluence angle also induces the increase in the transverse dispersion due to the maintenance of the strong transverse velocity shear, as shown in Figure 7. In particular, the effects of confluence angle decrease were relevant to the higher discharge ratio. For example, the increase of transverse mixing by the confluence angle decrease was insignificant in Case Q1, but the transverse mixing increase was noticeable in Case Q6.

Another notable feature is that the dosage curves show a local peak near *η* =1 in *x* = *W* for α = 45° and 60°. The local growth of θ/Θ indicates that the amount of solute trapped in the recirculation zone is released along the inner bank. However, these properties have not appeared in the results for α = 90°. The shear layer formed at the recirculation zone boundary is created by drawing a larger radius at α = 90° than α = 45° (see Figure 5a,c). Thus, for α = 90°, the solute flow turns around the recirculation zone, and the solute is mainly introduced into the recirculation zone by the circular cell inside the mixing interface. Conversely, for α = 45° and 60°, the solute flows along the downstream junction corner by the tributary flow direction and then inflows to the recirculation zone. 

The transverse dispersion coefficient (*D_T_*) was calculated using the STMM (Equation (7)) from the dosage curves obtained from *x* = *W* to *x* = 7*W*. Table 3 shows the calculation results of the dimensionless transverse dispersion coefficient ($D_{T}/hu^{*}$), where $u^{*}=\sqrt{gR_{h}^{7/3}{\left(nU\right)}^{2}}$ is the shear velocity and $R_{h}$ is the hydraulic radius. The results show that the transverse dispersion coefficient increases as the discharge ratio increase and the confluence angle decrease. For the results of α = 60° and 90°, $D_{T}/hu^{*}$ was 0.063–0.216, corresponding to the transverse dispersion coefficient range usually observed in straight channels [13]. The transverse dispersion increases between α = 60° and 90° was insignificant for the discharge ratio less than 1.0 as in Case Q1, Q2, and Q3. For the results of α = 45° and *M*_r_ > 1, the transverse dispersion noticeably increases compared to the other two cases. $D_{T}/hu^{*}$ was 0.39–0.67, which are included in the transverse dispersion coefficient range for meandering channels [13].

**Figure 11:**
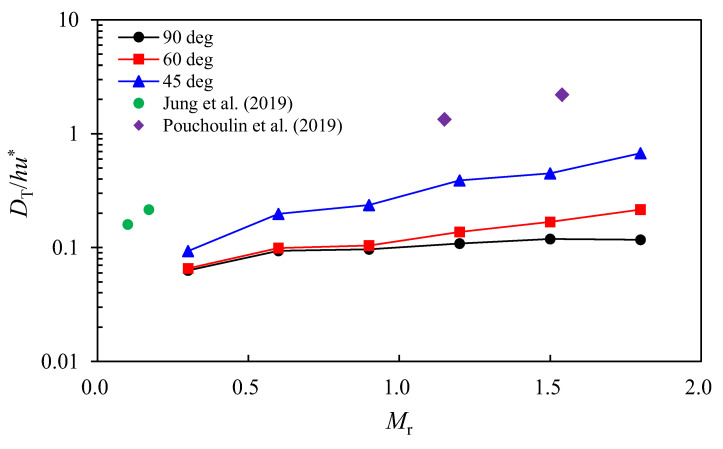
Change of the transverse dispersion coefficient according to the momentum ratio [8,21].

The inflow of high momentum tributary in a small confluence angle promotes to increase the transverse dispersion coefficient. As shown in Figure 7, the vertical shear of the transverse velocity produced by the circular motion increases with the high momentum ratio and persists further downstream for the small confluence angle. Thus, the momentum ratio and confluence angle influence the transverse dispersion coefficient according to the theoretical derivation shown in Equation (5). To figure out these relations, $D_{T}/hu^{*}$ was plotted against the momentum ratio (*M*_r_) with changing confluence angles in Figure 11. The results show that $D_{T}/hu^{*}$ increases with the increase of *M*_r_ while the value decreases at higher confluence angles. The increase rate of $D_{T}/hu^{*}$ against *M*_r_ increases as the confluence angle decreases. The results were compared with the field measurements [8,21] in which the $D_{T}/hu^{*}$ was obtained in low angle confluence (less than 30°). The field measurement results support the results of this study, in which the transverse mixing is activated as the increase of *M*_r_ and the decrease of the confluence angle. Additional distinctive features are that $D_{T}/hu^{*}$ from the field measurements increased as *M*_r_ increased, as also shown in this study.

## 4. Discussions

The simulation results show that the change of momentum and confluence angle influences the flow structure associated with mixing properties in the channel confluence. The complex interrelationships in flow structures and solute dispersion induce difficulties in understanding the impact of the inflow of contaminated tributaries on the downstream area. Thus, in this study, the simplified geometry condition, which was the rectangular cross-section and the concordant beds, was used to focus on the effects of flow structures on the transverse dispersion. Table 4 shows a summary of the flow and mixing characteristics according to the change in the momentum ratio and the confluence angle. In this table, three factors that affect the transverse dispersion are listed among several flow features in confluence. The tributary inflow collapses to the main channel flow and a downwelling flow occurs, which creates the mixing interface accompanying the clockwise-rotating helical motion. The change of transverse velocity structures influences the transverse shear dispersion, which is a more dominant mechanism than turbulent diffusion [13,22]. At the same time, the recirculation zone originating from the downstream junction corner plays a role as a storage zone, which traps contaminated water and elongates the tail of concentration.

The recirculation zone size is known to be enlarged with the increase in the momentum ratio and confluence angle from experimental and simulation results [9,31,32,34]. The enlarged recirculation zone drives to increase the retention time of the trapped solute cloud, and thus, the pollutant is trapped and accumulates at the inner bank at a larger confluence angle (Figure 10b,c). However, the high momentum ratio reduces the solute trapping in the storage with the high vertical deviations of transverse velocity shear that extracts solute from the recirculation zone. The tributary inflow with high momentum increases the stream-wise flow velocity and simultaneously steers the alignment of the mixing interface to the outer bank (Figure 5). The decrease of transverse momentum due to the converging two streams at the mixing interface produces the clockwise-rotating helical motion [5]. The high momentum tributary intensifies the helical motion, and the intensity of transverse velocity shear increases inside the mixing layer. For the larger confluence angle, the transverse velocity shear decreases rapidly as the flow becomes farther from the confluence. The persistence of transverse velocity shear over flow distance increases the transverse dispersion. Thus, the transverse dispersion associated with the vertical shear of transverse velocity decreases in a larger confluence angle and increases in the high momentum ratio.

**Figure 12:**
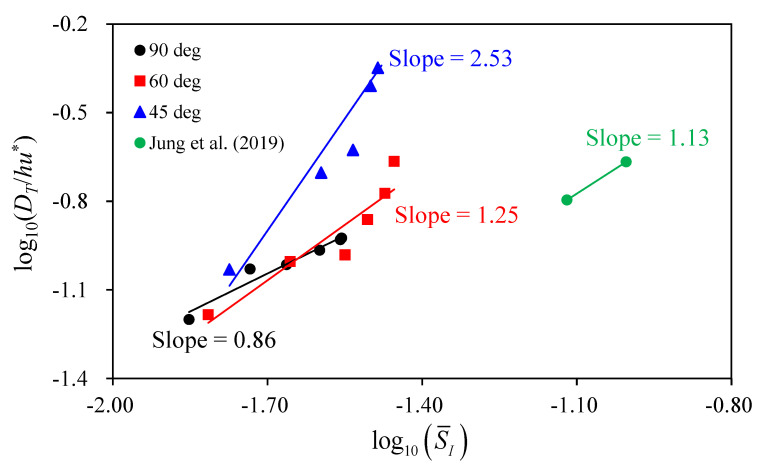
Relations of ${\bar{S}}_{I}$ and dimensionless transverse dispersion coefficient [21].

The vertical deviations of transverse velocity contribute to increase the transverse dispersion. The relation between the vertical deviations of transverse velocity and the transverse dispersion were evaluated using the secondary flow intensity ($S_{I}$). Figure 12 shows variations of the transverse dispersion coefficient against the variations of ${\bar{S}}_{I}$, which are the averaged values from *x* = *W* to *x* = 7*W*. The results show that $D_{T}/hu^{*}$ exponentially increases, corresponding to the increase of ${\bar{S}}_{I}$. From the simulation results, the increase rate of $D_{T}/hu^{*}$ (slope in Figure 12) increases with the larger confluence angle, in which the slope is 0.86 for α = 90° and 2.53 for α = 45°. However, $D_{T}/hu^{*}$ obtained from the Nakdong River in South Korea has the slope of 1.13, even though the confluence angle is smaller than 30° and low momentum ratio (*M*_r_ = 0.1–0.17) [21]. The study area shown by Jung et al. [21] has a curvature downstream of the confluence. The curvature created the second kind of secondary flow and produced a strong secondary flow intensity compared to the straight channel modeled in this study. These results indicate that the high momentum ratio and small confluence angle promote to increase in the transverse dispersion downstream of the confluence. However, the channel curvature accelerating the strong secondary flow intensity has a more dominant effect on the transverse dispersion coefficient increase.

## 5. Conclusions

This paper investigated the complex interrelationship between the flow structures and shear dispersion in confluence from the numerical simulation results. Pollutants introduced from the tributary were dispersed in transverse direction by the vertical shear of transverse velocity, and the contaminants were trapped in the recirculation zone by the helical flow created by the convergence of the mainstream and tributary. The related findings of this research are summarized as follows:

1. The tributary with high momentum created a strong helical motion in the cross-section downstream of the confluence and led to the inflow of pollutants to the recirculation zone. Simultaneously, the strong vertical deviations of transverse velocity due to the high momentum tributary contributed to the discharge of the pollutants from the recirculation zone. Thus, the retention time of pollutants in the recirculation zone decreased with the increase in the momentum ratio.
2. The flow structures induced by the large confluence angle contributed to increasing both the recirculation zone size and the retention time of pollutants. However, the larger flow radius generated from the downstream junction corner for α = 90° than that of other confluence angles reduced the pollutant accumulation flowing into the recirculation zone.
3. The strong secondary flow intensity generated from the high momentum tributary promoted the transverse dispersion coefficient to increase with the persistence of helical motion. However, the helical motion persistence rapidly decreased as flowing downstream and led to a decrease in the transverse dispersion for the large confluence angle.

The results from this study helped to broaden knowledge in evaluating the environmental effects of contaminated tributary inflows. From the interrelationship between the hydrodynamic properties and transverse dispersion downstream confluence, we were able to conduct an analysis which could be applied to future efficient water quality management. However, the results shown in this study have some limitations that the flow structures resulted in the RANS model cannot reproduce the streamwise-oriented vortical cells that appeared in the mixing interface due to underestimation of the secondary flows in confluences [4]. Even with these limitations, this study provides significant results on the influences of the momentum ratio and confluence angle on the reach scale transverse dispersion, which were investigated to enhance the knowledge of the complex interrelationships of those phenomena.

## Figures and Tables

**Table 1:** Simulation conditions for flow and mass transport models.

Case	*Q*_m_ (m^3^/s)	*Q*_b_ (m^3^/s)	*h_d_* (m)	*C*_0_ (ppm)	*dt* (s)	*C*_s_	Manning’s *n*	*Q*_r_	*M*_r_	Fr_d_
Q1	17.03	9.33	3.34	100	0.01	0.2	0.013	0.35	0.3	0.14
Q2		13.19				0.2		0.44	0.6	0.16
Q3		16.15				0.2		0.49	0.9	0.17
Q4		18.65				0.3		0.52	1.2	0.19
Q5		20.85				0.3		0.55	1.5	0.20
Q6		22.84				0.3		0.57	1.8	0.21

**Table 2:** Statistical properties of *C-t* curves at *x* = *l*_s_/2, *y* = *b_s_*/2 (*C_p_*: peak concentration; *t_p_*: time for occurrence of *C*_p_; *μ_t_*: centroid of the *C-t* curve; *t_d_*: retention time of the contaminated water; *ξ_t_*: Skewness coefficient).

*α* (°)	Case	*C*_p_ (ppm)	*t*_p_ (sec)	*μ*_t_ (sec)	*t*_d_ (sec)	*ξ*_t_
90	Q1	22.9	348.6	380.9	520	0.81
	Q2	30.6	308.6	328.2	405	0.80
	Q3	32.9	283.6	305.8	375	0.71
	Q4	37.2	273.6	288.6	345	0.66
	Q5	39.8	263.6	278.1	320	0.62
	Q6	56.6	328.6	327.3	380	0.20
60	Q1	42.9	314.0	338.1	405	1.20
	Q2	47.7	268.6	296.1	355	1.10
	Q3	49.7	258.6	280.0	330	1.02
	Q4	51.9	248.6	267.4	310	0.97
	Q5	54.0	238.6	256.9	295	0.94
	Q6	55.7	234.0	334.8	285	0.90
45	Q1	39.4	293.6	320.9	350	1.22
	Q2	42.3	263.6	285.2	310	1.08
	Q3	53.3	253.6	271.4	300	0.91
	Q4	53.7	243.6	261.6	290	0.85
	Q5	55.5	233.6	251.4	280	0.83
	Q6	57.0	229.0	330.3	270	0.77

**Table 3:** Estimation results of transverse dispersion coefficient according to momentum ratio and confluence angle.

Case	$D_{T}/hu^{*}$					
	Q1 (*M*_r_ = 0.3)	Q2 (*M*_r_ = 0.6)	Q3 (*M*_r_ = 0.9)	Q4 (*M*_r_ = 1.2)	Q5 (*M*_r_ = 1.5)	Q6 (*M*_r_ = 1.8)
α = 90°	0.063	0.093	0.097	0.108	0.119	0.117
α = 60°	0.066	0.099	0.104	0.137	0.169	0.216
α = 45°	0.093	0.198	0.236	0.390	0.448	0.674

**Table 4:** Effects of the confluence angle and momentum ratio to flow and mixing properties in confluence.

	Factors	Increase of Confluence Angle	Increase of Momentum Ratio
Flow structure	Recirculation zone size (*l*_s_ and *b*_s_)	Increase	Increase
	Mixing interface alignment	Not influenced	Created to the outer bank
	Intensity of vertical deviations of transverse velocity $\left(\sqrt{{\bar{v^{\prime }}}^{2}}/U\right)$	Decrease as flowing downstream	Increase
Mixing properties	Retention time in recirculation zone (*t*_d_)	Increase	Decrease
	Transverse dispersion coefficient $\left(D_{T}/hu^{*}\right)$	Decrease	Increase

## Data Availability

The data presented in this study will be shared by the authors if requested.

## References

[1] Lewis Q., Rhoads B., Sukhodolov A., Constantinescu G. (2019). Advective lateral transport of streamwise momentum governs mixing at small river confluences. Water Resour. Res..

[2] Gualtieri C., Ianniruberto M., Filizola N. (2019). On the mixing of rivers with a difference in density: The case of the Negro/Solimões confluence, Brazil. J. Hydrol..

[3] Yuan S., Tang H., Xiao Y., Qiu X., Zhang H., Yu D. (2016). Turbulent flow structure at a 90-degree open channel confluence: Accounting for the distortion of the shear layer. J. Hydro-Environ. Res..

[4] Constantinescu G., Miyawaki S., Rhoads B., Sukhodolov A., Kirkil G. (2011). Structure of turbulent flow at a river confluence with momentum and velocity ratios close to 1: Insight provided by an eddy-resolving numerical simulation. Water Resour. Res..

[5] Constantinescu G., Miyawaki S., Rhoads B., Sukhodolov A. (2012). Numerical analysis of the effects of momentum ratio on the dynamics and sediment-entrainment capacity of coherent flow structures at a stream confluence. J. Geophys. Res..

[6] Rhoads B.L., Kenworthy S.T. (1995). Flow structure at an asymmetrical stream confluence. Geomorphology.

[7] Lane S.N., Parsons D.R., Best J.L., Orfeo O., Kostaschuk R.A., Hardy R.J. (2008). Causes of rapid mixing at a junction of two large rivers: Rio Parana and Rio Paraguay, Argentina. J. Geophys. Res..

[8] Pouchoulin S., Coz J.L., Mignot E., Gond L., Riviere N. (2019). Predicting transverse mixing efficiency downstream of a river confluence. Water Resour. Res..

[9] Shin J., Lee S., Park I. (2021). Analysis of storage effects in the recirculation zone based on the junction angle of channel confluence. Appl. Sci..

[10] Chabokpour J., Azamathulla H.M. (2022). Numerical simulation of pollution transport and hydrodynamic characteristics through the river confluence using FLOW3D. Water Supply.

[11] Albers C., Steffler P. (2007). Estimating transverse mixing in open channels due to secondary current-induced shear dispersion. J. Hydraul. Eng..

[12] Baek K.O., Seo I.W. (2011). Transverse dispersion caused by secondary flow in curved channels. J. Hydraul. Eng..

[13] Rutherford J.C. (1994). River Mixing.

[14] Jeon T.M., Baek K.O., Seo I.W. (2007). Development of an empirical equation for the transverse dispersion coefficient in natural streams. Environ. Fluid Mech..

[15] Seo I.W., Choi H.J., Kim Y.D., Han E.J. (2016). Analysis of two-dimensional mixing in natural streams based on transient tracer tests. J. Hydraul. Eng..

[16] Baek K.O., Seo I.W. (2017). Estimation of the transverse dispersion coefficient for two-dimensional models of mixing in natural stream. J. Hydro-Environ. Res..

[17] Lewis Quinn W., Bruce L. (2015). Rhoads. Rates and patterns of thermal mixing at a small stream confluence under variable incoming flow conditions. Hydrol. Process..

[18] Constantinescu G., Miyawaki S., Rhoads B., Sukhodolov A. (2016). Influence of planform geometry and momentum ratio on thermal mixing at a stream confluence with a concordant bed. Environ. Fluid Mech..

[19] Sukhodolov A.N., Sukhodolova T.A. (2019). Dynamics of flow at concordant gravel bed river confluences: Effects of junction angle and momentum flux ratio. J. Geophys. Res. Earth Surf..

[20] Rhoads B.L., Sukhodolov A.N. (2001). Field investigation of three-dimensional flow structure at stream confluences: 1. Thermal mixing and time-averaged velocities. Water Resour. Res..

[21] Jung S.H., Seo I.W., Kim Y.D., Park I. (2019). Feasibility of velocity-based method for transverse mixing coefficients in river mixing analysis. J. Hydraul. Eng..

[22] Fischer H.B., List J.E., Koh R.C.Y., Imberger J., Brooks N.H. (1979). Mixing in Inland and Coaster Waters.

[23] Taylor G.I. (1954). The dispersion of matter in turbulent flow through a pipe. Proc. R. Soc. London. Ser. A. Math. Phys. Sci..

[24] Beltaos S. (1980). Transverse mixing tests in natural streams. J. Hydraul. Div..

[25] Baek K.O. (2018). Flowchart on choosing optimal method of observing transverse dispersion coefficient for solute transport in open channel flow. Sustainability.

[26] Hamrick J.M. (1992). A three-dimensional environmental fluid dynamics computer code: Theoretical and computational aspects. Special Report in Applied Marine Science and Ocean Engineering, 317.

[27] Weber L.J., Eric D.S., Nicola M. (2001). Experiments on flow at a 90° open-channel junction. J. Hydraul. Eng..

[28] Yang Q.Y., Wang X.Y., Lu W.Z., Wang X.K. (2009). Experimental study on characteristics of separation zone in confluence zones in rivers. J. Hydrol. Eng..

[29] Yu Q., Yuan S., Rennie C.D. (2019). Experiments on the morphodynamics of open channel confluences: Implications for the accumulation of contaminated sediments. J. Geophys. Res. Earth Surf..

[30] Best J.L., Reid I. (1984). Separation zone at open-channel junctions. J. Hydraul. Eng..

[31] Gurram S.K., Karki K.S., Hager W.H. (1997). Subcritical junction flow. J. Hydraul. Eng..

[32] Shakibainia A., Tabatabai M.R.M., Zarrati A.R. (2010). Three-dimensional numerical study of flow structure in channel confluences. Can. J. Civ. Eng..

[33] Penna N., Marchis M.D., Canelas O.B., Napoli E., Cardoso A., Gaudio R. (2018). Effect of the junction angle on turbulent flow at a hydraulic confluence. Water.

[34] Huang J., Weber L.J., Lai Y.G. (2002). Three-dimensional numerical study of flows in open-channel junctions. J. Hydraul. Eng..

